# Diagnostic Reasoning by Expert Clinicians: What Distinguishes Them From Their Peers?

**DOI:** 10.7759/cureus.19722

**Published:** 2021-11-18

**Authors:** Bharat Kumar, Kristi Ferguson, Melissa Swee, Manish Suneja

**Affiliations:** 1 Rheumatology, University of Iowa Hospitals & Clinics, Iowa City, USA; 2 Medical Education, University of Iowa Carver College of Medicine, Iowa City, USA; 3 Nephrology, University of Iowa Hospitals & Clinics, Iowa City, USA; 4 Internal Medicine, University of Iowa Hospitals & Clinics, Iowa City, USA

**Keywords:** general internal medicine, internal medicine, diagnostic decision-making, continuing medical education, diagnostic reasoning

## Abstract

Objectives

Expert clinicians (ECs) are defined in large part as a group of physicians recognized by their peers for their diagnostic reasoning abilities. However, their reasoning skills have not been quantitatively compared to other clinicians using a validated instrument.

Methods

We surveyed Internal Medicine physicians at the University of Iowa to identify ECs. These clinicians were administered the Diagnostic Thinking Inventory, along with an equivalent number of their peers in the general population of internists. Scores were tabulated for structure and thinking, as well as four previously identified elements of diagnostic reasoning (data acquisition, problem representation, hypothesis generation, and illness script search and selection). We compared scores between the two groups using the two-sample t-test.

Results

Seventeen ECs completed the inventory (100%). Out of 25 randomly-selected non-EC internists (IM), 19 completed the inventory (76%). Mean total scores were 187.2 and 175.8 for the EC and the IM groups respectively. Thinking and structure subscores were 91.5 and 95.71 for ECs, compared to 85.5 and 90.3 for IMs (p-values: 0.0783 and 0.1199, respectively). The mean data acquisition, problem representation, hypothesis generation, and illness script selection subscores for ECs were 4.46, 4.57, 4.71, and 4.46, compared to 4.13, 4.38, 4.45, and 4.13 in the IM group (p-values: 0.2077, 0.4528, 0.095, and 0.029, respectively).

Conclusions

ECs have greater proficiency in searching for and selecting illness scripts compared to their peers. There were no statistically significant differences between the other scores and subscores. These results will help to inform continuing medical education efforts to improve diagnostic reasoning.

## Introduction

In recent years, the term “expert clinician” (EC) has been used to define physicians from any specialty or subspecialty who have attained high levels of proficiency in a variety of skills considered essential to clinical practice and education [1]. Although the specific definition has remained elusive, ECs are acknowledged to be superior diagnosticians from whom trainees can learn valuable lessons [2]. For that reason, “Expert clinician” and “Master clinician” programs have been established at sites like the University of Iowa. This study aims to distinguish elements of diagnostic reasoning that characterize ECs compared to peers, using the previously validated Diagnostic Thinking Inventory (DTI) [3].

## Materials and methods

This study was approved by the Institutional Review Board at the University of Iowa. The investigation was split into three parts.

First, each question in the DTI was classified into one of four categories: data acquisition, problem representation, hypothesis generation, and illness script search and selection. Definitions for these four steps of diagnostic reasoning were adopted from Bowen and colleagues [4]. The wording of each question was scrutinized independently by two investigators (BK and MS), and results were compared. Two rounds of reconciliation were pursued in order to obtain a consensus classification of these questions. 

Secondly, ECs were identified through a survey of all Internal Medicine physicians at the University of Iowa. Inclusion criteria were permanent faculty status, appointment within the Department of Internal Medicine, and over 50% clinical effort. Exclusion criteria were adjunct or visitor status, and research or administrative effort greater than 50%. They were asked to identify one or more clinicians amongst themselves who are considered ECs based on their diagnostic skills. Those nominated by at least five colleagues were designated as ECs. Once identified, they were administered the DTI. 

Finally, a third investigator (KF) used a random number generator to identify a sample of 25 internists not recognized as ECs (IM) who were then administered the DTI. Demographic information about both the EC and the IM groups was also obtained, including years in practice, age, under-represented minority status, international medical graduate status, and residency location. For binary variables, the Fisher exact test was used for comparison while for continuous variables, unpaired t-tests were used.

Results were tabulated and uploaded into SAS® (SAS Institute, Cary, North Carolina). Descriptive statistics, including mean, median, and variance were calculated for the scores and subscores. The two-sample t-test was employed to compare means between the EC and IM groups.

## Results

The DTI was split into four categories as given in the Materials and Methods section, composed of eight to 14 questions each (Table 1). There was agreement among the two investigators for 38 out of 41 at the first round, and 41/41 by the second round for reconciliation.

**Table 1:** Classification of items in the DTI Items from the DTI were classified by the aspect of diagnostic reasoning and the element of diagnostic reasoning [3]. DTI: Diagnostic Thinking Inventory

Item											Aspect of Diagnostic Reasoning	Element of Diagnostic Reasoning
1. When the patient presents his symptoms,	I think of the symptoms in the precise words used by the patient		O	O	O	O	O	O		I think of the symptoms in more abstract terms than the expressions actually used (e.g. ‘4 days duration’; becomes ‘acute’; two hands become bilateral)	Structure	Problem Representation
2. In considering each diagnosis,	I try to evaluate their relative importance		O	O	O	O	O	O		I try to give them equal importance or weighting	Thinking	Search for and Selection of Illness Scripts
3. In thinking of diagnostic possibilities,	I think of these possibilities early on in the case		O	O	O	O	O	O		first I collect the clinical information and then I think about it	Thinking	Hypothesis Generation
4. When I am interviewing a patient,	I often seem to get one idea stuck in my mind about what might be wrong		O	O	O	O	O	O		I usually find it easy to explore various possible diagnoses	Thinking	Hypothesis Generation
5. Throughout the interview,	if I follow the patients line of thought, I tend to lose my own thread		O	O	O	O	O	O		I can still keep my own ideas clear even if I follow the patient’s line of thought	Thinking	Hypothesis Generation
6. When it comes to making up my mind about a diagnosis,	I do not mind postponing my diagnostic decisions about a case		O	O	O	O	O	O		I feel obliged to go for one diagnosis or another even if I am not very certain	Thinking	Hypothesis Generation
7. Once a patient has clearly presented his symptoms and signs,	I think about them in my mind in the patient’s own words		O	O	O	O	O	O		I translate them in my mind into medical terms (e.g. numbness becomes paraesthesia)	Structure	Problem Representation
8. In relation to the routine history,	I often feel I did not cover the routine history		O	O	O	O	O	O		I usually cover the routine history to my satisfaction	Structure	Data Acquisition
9. As the patient tells his story and the case unfolds,	I often find it difficult to remember what has been said		O	O	O	O	O	O		I can usually keep track in my mind what has been said	Structure	Data Acquisition
10. During the course of the interview, I find that,	some key pieces of information seem to leap out at me		O	O	O	O	O	O		it is often difficult to know which items of information to latch on to	Structure	Problem Representation
11. When I cannot make sense of the patients symptoms,	I move on and gather new information to trigger new ideas		O	O	O	O	O	O		I ask the patient to define those symptoms more clearly	Thinking	Data Acquisition
12. In considering diagnostic possibilities,	I often come up with unlikely diagnoses		O	O	O	O	O	O		I am usually in the right area	Structure	Hypothesis Generation
13. While I am collecting information about a patient,	the various items of information usually seem to group themselves together in my mind		O	O	O	O	O	O		I often have difficulty in seeing how the pieces of information relate to each other	Structure	Problem Representation
14. When the diagnosis becomes known and I realize I have missed it initially,	it is often because I knew the disease but failed to think about it		O	O	O	O	O	O		it is often because I did not know enough about the disease	Structure	Search for and Selection of Illness Scripts
15. During the clinical interview,	I cannot bring myself to dismiss some information as irrelevant		O	O	O	O	O	O		I am quite happy to dismiss some information as irrelevant	Thinking	Problem Representation
16. When I cannot make sense of the patients symptoms and signs,	I move on to get new information and a new perspective		O	O	O	O	O	O		I look at them from a different perspective before moving on	Thinking	Data Acquisition
17. When I consider a number of possible diagnoses,	the diagnoses tend to be related to one another		O	O	O	O	O	O		the diagnoses tend to be scattered	Structure	Hypothesis Generation
18. When a possible diagnosis comes to mind,	I usually find myself anticipating possible abnormal signs and symptoms that go with that diagnosis		O	O	O	O	O	O		quite often, it does not help me decide what to ask the patient next	Structure	Hypothesis Generation
19. When I know very little about a particular type of disease,	I can still usually come up with a diagnosis		O	O	O	O	O	O		I have great difficulty in reaching a diagnosis	Structure	Search for and Selection of Illness Scripts
20. In considering the patient’s signs and symptoms,	I think of them in absolute terms as stated by the patient		O	O	O	O	O	O		I think of them in terms of possible opposites (e.g. progressive vs. sudden; unilateral vs. bilateral; spastic vs. flaccid)	Structure	Problem Representation
21. When I know a lot about a particular type of disease and have to make a diagnosis,	I find it relatively easy to pin down a diagnosis		O	O	O	O	O	O		I often seem to be all over the place and have difficulty in pinning down a diagnosis	Structure	Search for and Selection of Illness Scripts
22. As the history progresses and I already have some idea about the possible diagnosis(es)	new information often makes me have more ideas		O	O	O	O	O	O		new information does not make me have more ideas	Structure	Hypothesis Generation
23. When I am taking a history, I find that,	I can get new ideas just by going over the existing information in my mind		O	O	O	O	O	O		I need to have new information to make me have a new idea about the case	Thinking	Hypothesis Generation
24. When the patient uses imprecise or ambiguous expressions,	I let him/her go on to maintain the flow of the interview		O	O	O	O	O	O		I make him/her clarify precisely what he means before going on	Thinking	Data Acquisition
25. After an interview with a patient,	I rarely think of other things that I should have asked in relation to the patients disorder		O	O	O	O	O	O		I often think of other things I should have asked in relation to the patients disorder	Structure	Data Acquisition
26. When a piece of information comes along and makes me think of a possible diagnosis,	it makes me go back to the previous information to see if things fit together or not		O	O	O	O	O	O		it rarely makes me review the information I gathered previously	Thinking	Hypothesis Generation
27. In relation to the diagnosis I eventually make,	I usually have very few doubts		O	O	O	O	O	O		I often feel too uncertain for my own comfort	Thinking	Search for and Selection of Illness Scripts
28. In making a diagnostic decision,	I decide by considering each diagnosis separately on its own merits		O	O	O	O	O	O		I decide by comparing and contrasting the possible diagnoses	Thinking	Search for and Selection of Illness Scripts
29. When I know a lot about a particular type of disease and have to make a diagnosis,	I check up on most possibilities before reaching a decision		O	O	O	O	O	O		I often have lots of ideas that I don’t explore further	Structure	Search for and Selection of Illness Scripts
30. As the case unfolds,	I do not find it useful to summarize as I go along		O	O	O	O	O	O		I periodically take stock of the data and my ideas	Thinking	Problem Representation
31. When I reach my diagnostic decisions,	there is often left-over information I have just forgotten about		O	O	O	O	O	O		I usually will have considered all the information	Structure	Data Acquisition
32. When I have got an idea about what might be wrong with a patient,	I feel most comfortable if I can follow it up without being diverted		O	O	O	O	O	O		I feel happy to go off on another track and come back to my original ideas later	Thinking	Hypothesis Generation
33. When I come up with a broad idea as to what might be wrong with the patient,	I can usually proceed to a specific diagnosis		O	O	O	O	O	O		I find it difficult to put it into specific terms	Structure	Problem Representation
34. Throughout the interview,	I manage to test my ideas even if I let the patient control the interview		O	O	O	O	O	O		I am only successful if I can control the direction of the interview	Thinking	Data Acquisition
35. In relation to choosing from among the diagnostic ideas I have,	I am usually not capable of wholly ruling out any of the ideas I have had		O	O	O	O	O	O		I am capable of ruling out most of my ideas completely	Thinking	Search for and Selection of Illness Scripts
36. Once I have made my mind up about a patient,	I am prepared to change my mind		O	O	O	O	O	O		I really do not like to change my mind	Thinking	Hypothesis Generation
37. When I consider my diagnostic ideas I do so on the basis of,	On the case as a whole so far		O	O	O	O	O	O		A few outstanding symptoms and signs	Structure	Hypothesis Generation
38. If I do not know what to make of a clinical interview,	I can readily see the information in new ways		O	O	O	O	O	O		I find it difficult to see the information in new ways	Thinking	Data Acquisition
39. When I order laboratory tests,	I do it as part of the routine clinical investigation		O	O	O	O	O	O		I do it expecting specific information or supporting evidence	Structure	Hypothesis Generation
40. In considering diagnostic possibilities,	I compare and contrast the possible diagnoses		O	O	O	O	O	O		I consider each diagnosis separately on its own merits	Thinking	Search for and Selection of Illness Scripts
41. In terms of the way I conduct an interview,	I usually cover the ground that I need to during the interview		O	O	O	O	O	O		Quite often I do not ask all the questions I should do at the time	Thinking	Data Acquisition

Surveys asking for the names for ECs were distributed to the Internal Medicine physicians (n=82), of which 78 replied (95.1%). Seventeen ECs were identified through this process. Of the remaining 65 internists, 25 (38.5%) non-ECs (IM) received the DTI, of which 19 completed the questionnaires (76%). 

Using the Fisher's exact test, there was no statistically significant difference between the EC group and the IM group, respectively, when comparing gender (52.9% male vs. 47.3% male, p=1.00), under-represented minority status (17.6% vs. 21.1%, respectively, p=1.00), residency location (35.3% at Iowa vs. 42.1% at Iowa, p=1.00), international medical graduate status (17.6% vs. 36.8%, 0.271), and MD/PhD status (29.4% vs. 10.5%, respectively, p=0.2185). Using the unpaired t-test, there was no statistically significant difference in mean age in years (56.3+11.1 vs. 48.1+13.2, respectively, p=0.0531), but there was with respect to mean years in practice (25.4+6.4 vs. 21.3+5.3, respectively, p=0.0431).

**Figure 1:**
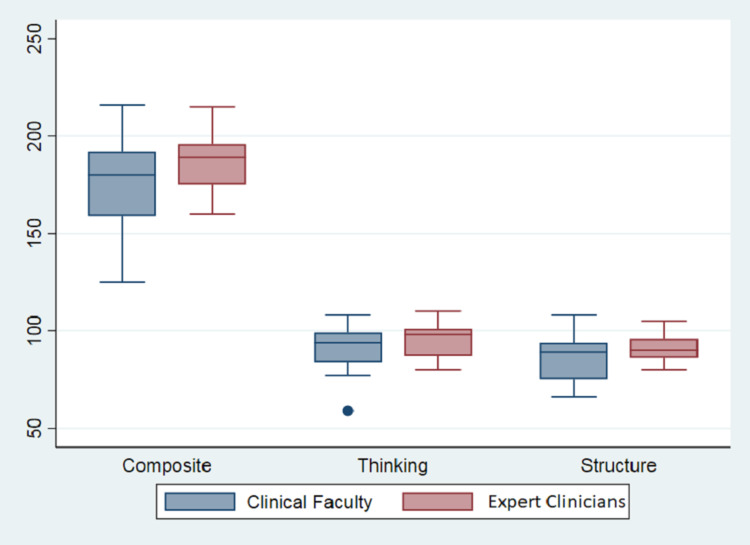
Distribution of DTI subscores based on the elements of diagnostic reasoning show the minimum, first quartile, median, third quartile, and maximum values for both the EC as well as the IM groups EC: expert clinicians; IM: non-expert internal medicine physicians; DTI: Diagnostic Thinking Inventory

ECs exhibited a higher total mean score (187.2), thinking subtotal (95.7), and structure subtotal (91.5) compared to the IM group, whose means were 175.8, 90.3, and 85.5, respectively (Figure 1). The standard deviations among ECs were lower for the total (14.6) as well as thinking and structure subtotals (8.6 and 7.1), compared to IMs (21.8, 11.4, and 11.8, respectively). The differences in means were not statistically significant (0.0783 for thinking and 0.1199 for structure).

**Figure 2:**
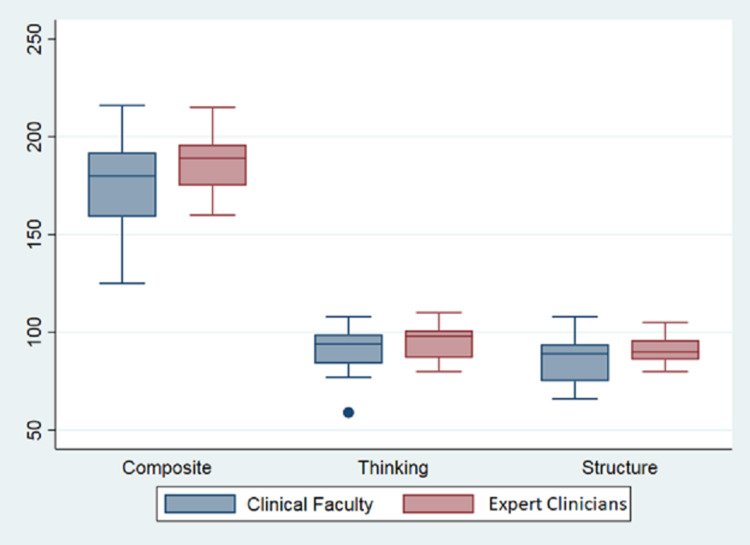
Distribution of DTI total scores, thinking subscores, and structure subscores show the minimum, first quartile, median, third quartile, and maximum values for both the EC and IM groups EC: expert clinicians; IM: non-expert internal medicine physicians; DTI: Diagnostic Thinking Inventory

When grouped by the elements of diagnostic reasoning, the ECs still had higher scores for all four elements, although this difference was statistically significant for only illness script selection, where the mean EC subscore was 4.46 (+0.31), vs. 4.13 (+0.53) in the IM group (p-value: 0.029). For data acquisition, problem representation, and hypothesis generation, subscore means (and associated standard deviations) were 4.13 (+0.93), 4.38 (+0.85), and 4.45 (+0.46) for the IM group, compared to 4.46 (+0.53), 4.57 (+0.56), and 4.71 (+0.47) for the EC group, respectively (Figure 2). The associated p-values were 0.2077, 0.4528, and 0.095, respectively (Table 2).

**Table 2:** DTI Item classification, mean values, and standard deviations The only element of diagnostic reasoning in which there is a statistically significant difference between EC and IM groups  is "Illness Script Search and Selection." EC: expert clinicians; IM: non-expert internal medicine physicians; DTI: Diagnostic Thinking Inventory

Element of Diagnostic Reasoning	Definition	Number of Pertinent Items	IM Mean (Standard Deviation)	EC Mean (Standard Deviation)	P-value
Data Acquisition	Elements of the history, the findings on physical examination, and the results of laboratory testing and imaging studies [3]	10	4.13 (0.93)	4.46 (0.53)	0.2077
Problem Representation	A one-sentence summary defining the specific case in abstract terms … illustrates the transformation of patient-specific details into abstract terms [3]	8	4.38 (0.85)	4.57 (0.56)	0.4528
Hypothesis Generation	The defining and discriminating clinical features of a disease, condition, or syndrome [3]	14	4.45 (0.46)	4.71 (0.47)	0.095
Illness Script Search and Selection	Conceptual models, such as groups of diseases, whereas others are representational memories of specific syndromes [3]	9	4.13 (0.52)	4.46 (0.31)	0.029
Structure	Availability of knowledge, stored in memory, during the diagnostic process. It is assumed that availability is a direct consequence of adequate knowledge organization [4]	20	90.3 (11.4)	95.71 (8.6)	0.1199
Thinking	The use of a variety of thinking means or processes that can be applied during the diagnostic process [4]	21	85.5 (11.8)	91.5 (7.1)	0.0783
Total		41	175.8 (21.8)	187.2 (14.6)	.0766

## Discussion

This study demonstrates that the diagnostic approach of ECs may be different than their peers. Specifically, ECs seem to be more proficient in searching and selecting for illness scripts. Illness scripts are defined as “conceptual models, such as groups of diseases [or] representational memories of specific syndromes [5].” Illness scripts are the result of experience and deliberate practice, suggesting that peer-recognized ECs continuously hone their understanding of key discriminating features, risk factors, and pathophysiologic mechanisms that define illness scripts [6]. This helps to explain findings from previously published literature that expert diagnosticians are able to diagnose conditions using relatively few pieces of clinical data [7]. It also reinforces the observations that ECs improve their diagnostic skills through continuous reflection [4, 8]. 

Of note, there were no statistically significant differences between the two groups with respect to gender, age, under-represented minority status, international medical graduate status, MD/PhD training, or residency location in Iowa. The mean years in practice was slightly higher among expert clinicians (25.4) compared to non-expert clinicians (21.3), but it is unclear how much this difference in seniority may impact diagnostic reasoning skills. 

Interestingly, there was no statistically significant difference in scores among the other elements of diagnostic reasoning. Hypothesis generation and data acquisition appeared to approach significance but were not significant at the p=0.05 significance level. Of note, illness script selection had the lowest score in both groups, suggesting that this is a more advanced skill to master, compared to the other three.

Likewise, there was no statistically significant difference between the "structure" and "knowledge" subscores in the DTI, which have been the two traditional categories used in prior analyses. Therefore, based on our analysis, it may be more appropriate to use this modified four-category breakdown to characterize the diagnostic reasoning process, using the same questions.

The strengths of our study include a robust prospective study design with high participation of staff physicians, including all of the identified ECs. The DTI has been validated as a tool to identify diagnostic reasoning skills [3]. The methodology by which the investigators categorized the questions was predetermined based on established definitions and criteria, enabling high rates of agreement after two rounds of reconciliation.

However, there are some notable limitations. The DTI is a self-administered test, so it is subject to social desirability biases. Also, this study was only conducted at one institution, the University of Iowa. However, the mean scores for the EC group have been higher than previously reported numbers for physicians in general, suggesting that they truly possess better diagnostic reasoning skills [8]. Finally, the numbers are relatively modest (36 total), which may explain why some of the other elements of diagnostic reasoning did not demonstrate statistical significance. While more participants may reduce the standard deviations, it is unclear how the means would change, particularly if the EC group were expanded. Similarly, our investigation also did not focus on how demographic features are correlated with DTI scores and therefore the lack of statistically significant differences between the two groups, with the exception of years in practice, may be due to an inadequate sample size to detect such differences. Lastly, the DTI was not designed to identify what aspects of illness script search and selection are most discriminating. Regardless, our data suggest that, in these four steps of diagnostic reasoning, the ability to search and select for illness scripts is the most specific marker of being a peer-recognized EC.

## Conclusions

ECs are recognized by peers in large part due to their diagnostic reasoning abilities. Compared to their peers in the general population of internists, ECs have greater proficiency in searching for and selecting illness scripts. This aligns well with prior observations that ECs engage in deliberate practice to build upon their prior experiences. Replication of these findings at other institutions may bolster such conclusions. Furthermore, these findings inform the development of EC and continuing medical education programs at other institutions.
